# Towards tailored teaching: using participatory action research to enhance the learning experience of Longitudinal Integrated Clerkship students in a South African rural district hospital

**DOI:** 10.1186/s12909-016-0607-3

**Published:** 2016-03-08

**Authors:** Klaus B. von Pressentin, Firdouza Waggie, Hoffie Conradie

**Affiliations:** Division of Family Medicine and Primary Care, Faculty of Medicine and Health Sciences, Stellenbosch University, PO Box 241, Cape Town, 8000 South Africa; Interdisciplinary Teaching and Learning Unit, Faculty of Community and Health Sciences, University of the Western Cape, Private Bag X17, Bellville, 7535 South Africa

**Keywords:** Educational activities, Clinical clerkship, District hospital, Teaching, Longitudinal clerkship, Workplace-based learning, Participatory action research

## Abstract

**Background:**

The introduction of Stellenbosch University’s Longitudinal Integrated Clerkship (LIC) model as part of the undergraduate medical curriculum offers a unique and exciting training model to develop generalist doctors for the changing South African health landscape. At one of these LIC sites, the need for an improvement of the local learning experience became evident. This paper explores how to identify and implement a tailored teaching and learning intervention to improve workplace-based learning for LIC students.

**Methods:**

A participatory action research approach was used in a co-operative inquiry group (ten participants), consisting of the students, clinician educators and researchers, who met over a period of 5 months. Through a cyclical process of action and reflection this group identified a teaching intervention.

**Results:**

The results demonstrate the gaps and challenges identified when implementing a LIC model of medical education. A structured learning programme for the final 6 weeks of the students’ placement at the district hospital was designed by the co-operative inquiry group as an agreed intervention. The post-intervention group reflection highlighted a need to create a structured programme in the spirit of local collaboration and learning across disciplines. The results also enhance our understanding of both students and clinician educators’ perceptions of this new model of workplace-based training.

**Conclusions:**

This paper provides practical strategies to enhance teaching and learning in a new educational context. These strategies illuminate three paradigm shifts: (1) from the traditional medical education approach towards a transformative learning approach advocated for the 21^st^ century health professional; (2) from the teaching hospital context to the district hospital context; and (3) from block-based teaching towards a longitudinal integrated learning model. A programme based on balancing structured and tailored learning activities is recommended in order to address the local learning needs of students in the LIC model. We recommend that action learning sets should be developed at these LIC sites, where the relevant aspects of work-place based learning are negotiated.

## Background

Since the first Longitudinal Integrated Clerkship (LIC) over 40 years ago, this medical education model has become recognised globally as an alternative to the traditional speciality-based block rotations [1]. According to the Consortium of Longitudinal Integrated Clerkships Research Collaborative, 45 LIC programmes exist around the world [2]. The LIC has gained recognition for producing medical doctors suitable for the generalist primary care environment [3, 4]. A recent World Health Organisation (WHO) report supported the need for training culturally sensitive and competent staff to attain and sustain universal health coverage (an ideal at the core of primary health care) [5]. The traditional Flexnerian approach to training doctors (by rotating students through specialist disciplines in larger tertiary hospitals, the so-called “traditional block model” of teaching) is not sufficient to meet the needs of 21st century communities [1, 6]. New graduates need to be competent in applying a patient-centred, integrated method of care, need to take cognisance of the social factors influencing illness patterns, and need to be equipped as ‘enlightened change agents’ [6]. These graduate attributes may be achieved through transformative learning, considered by the Lancet Commission on Education of Health Professionals for the 21st Century to be the highest of three successive levels of learning (compared to the informative and formative learning offered by previous reforms in health professions education) [6].

The LIC model is an example of instructional reform geared towards facilitating transformative learning [7]. The LIC has the potential to improve medical student teaching and learning in a time-efficient manner, develop a rural community of practice, strengthen the health system in which the students will work as doctors, and enable medical students to participate in the comprehensive care of patients over time [1–4, 7–11]. In a LIC, the students participate in the care of the same patients over time, develop learning relationships with the clinicians who care for these patients, and learn across all clinical disciplines simultaneously [7, 11]. International studies suggest that students who participate in LIC models perform better than or equivalent to the speciality-based rotations in terms of their clinical skills and knowledge acquisition. They also experience progressively higher levels of patient care responsibilities, demonstrate greater flexibility in addressing their own educational needs, have a positive view of educational continuity, sustain higher patient-centred attitudes, receive more feedback from faculty, and report more satisfaction with the curriculum [10–13].

In South Africa, Stellenbosch University (SU) offered the LIC as an alternative education model at the Ukwanda Rural Clinical School (RCS) for the first time in 2011. The RCS is situated in the Cape Winelands and Overberg Districts of the Western Cape, with the Worcester Regional Hospital at its centre. This regional facility provided specialist support to seven district hospitals and more than seventy clinics. An important component of the RCS was providing teaching and learning opportunities for students within the district health system. The RCS aimed to expose students in their training to rural health, real-life experiences, and clinical training over a longer period of time. They also intended to influence future career decisions to provide quality health care in rural communities [14].

The LIC at the RCS is designed for a unique South African context in which undergraduate medical students spend their sixth (final) year in the clinical team of a rural district hospital and its associated primary care platform [15]. Training takes place at district hospitals under the supervision of family physicians and the clinician team, who act as clinician educators for the students. This training occurs with support from general specialists at the regional hospital, as well as other health professionals. This integrated training at district level is a new approach for undergraduate medical students at SU, and is modelled on the international LIC experience, adapted to the SA context [16]. The curriculum has been adjusted, and extensive logistic arrangements have been made to support this LIC.

Robertson district hospital (one of the RCS’s LIC sites), hosted its first two groups of LIC students in 2012 (two students) and 2013 (three students). At the end of year 1 (2012), a discussion between the first group of LIC students and clinician educators at the Robertson LIC site expressed the need to enhance the students’ learning experience. Challenges experienced by students and clinician educators included learning across disciplines, tensions between academic textbook-learning and clinical learning activities, assessment-related anxiety (the LIC students have their final assessment at the tertiary academic institution) as well as staff uncertainty regarding their clinician educator responsibilities and the student learning outcomes. These challenges, typical of early student and clinician educator experiences when introducing the LIC into a new context [17–20], were also experienced at the other LIC sites of this RCS. A structured process was called for to improve the learning at the Robertson LIC site.

## Methods

### Study aim

This study aims to explore how to identify, design and implement a teaching and learning intervention to improve workplace-based learning for LIC students at a rural South African district hospital.

### Study design

**Fig. 1:**
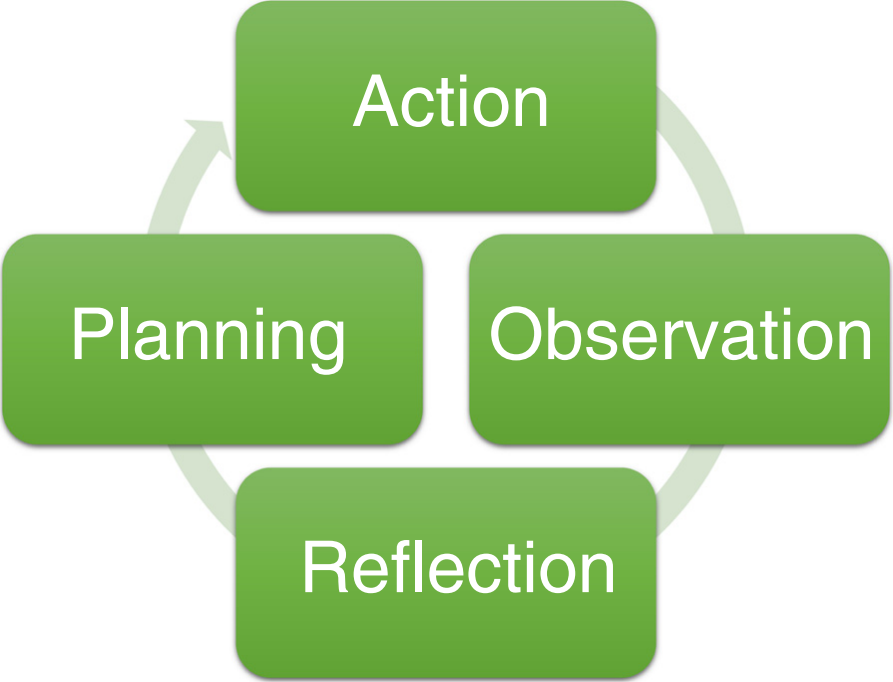
The action-reflection cycle (adapted from and used with permission of the authors [22])

A participatory action research (PAR) approach was chosen to generate a new understanding and possible solutions to the learning challenges experienced at this LIC site. This research method falls within the emancipatory-critical research paradigm, which allows researchers and participants to work together by reflecting on and changing their practice [21, 22]. A co-operative inquiry group (CIG) method was used, as it represents the professional tradition of PAR embraced by professionals from both the educational and health sectors, who wish to improve their practice (as opposed to the empowering and organisational traditions of PAR) [21]. The steps of the PAR cycle were followed (as illustrated in Fig. 1, adapted from and used with permission of Mash and Meulenberg‐Buskens [22]). This cyclical process follows four steps repeated in an ongoing spiral of action (having an experience), observation (reviewing the experience), reflection (learning from the experience) and planning (based on new learning) [21, 22].

### Setting

**Fig. 2:**
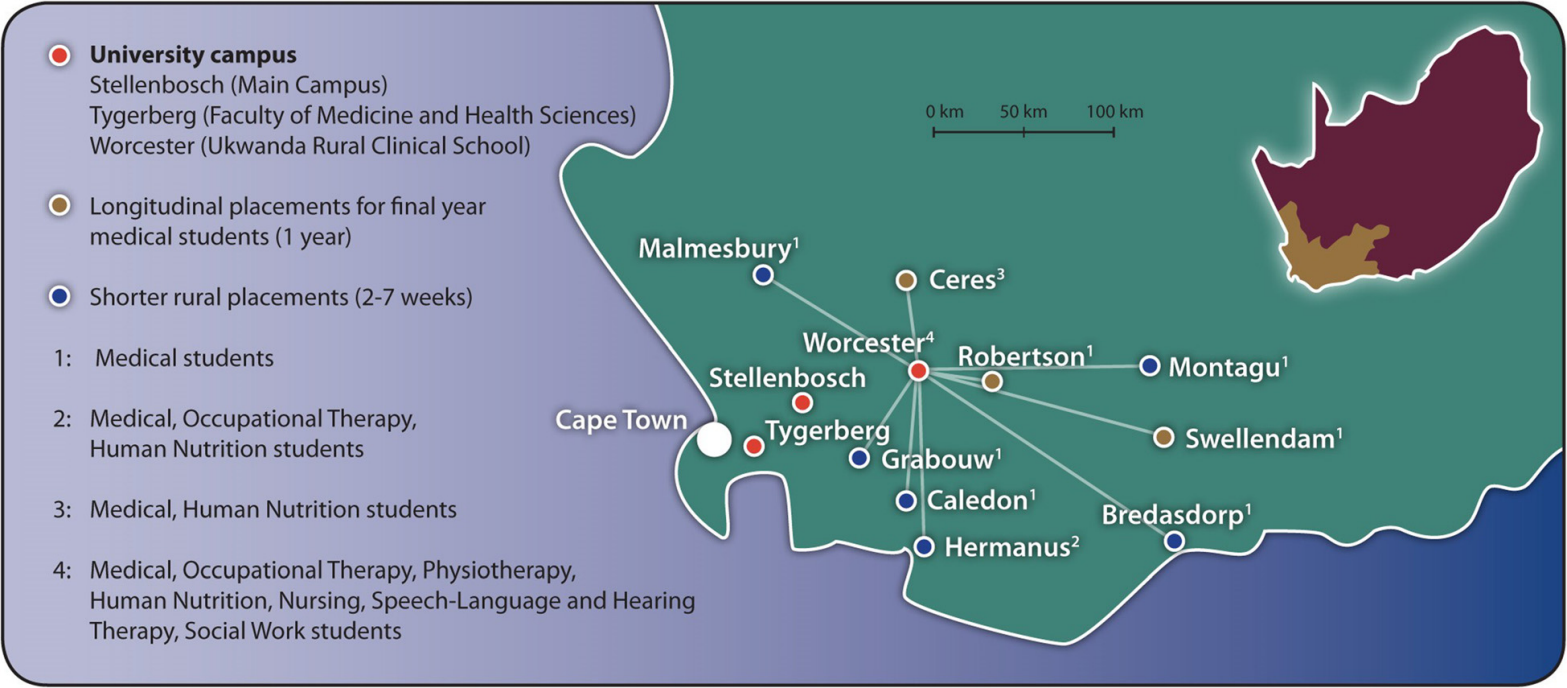
The Ukwanda Rural Clinical School of Stellenbosch University and the sites where students are placed (used with permission of the authors [23])

The study was conducted at Robertson District Hospital, which forms part of the Langeberg Sub-district of the Cape Winelands District in the Western Cape Province of South Africa. The Langeberg Sub-district serves a rural community of around 140 000 people and most of the population are dependent on the public health system. This sub-district’s health facilities include two district hospitals as well as seven fixed and three mobile clinics. The sub-district serves five towns (Robertson, Montagu, Bonnievale, Ashton and McGregor) and their surrounding farms. Robertson is approximately 50 kilometres from Worcester’s RCS and 160 kilometres from the Tygerberg medical campus (Faculty of Medicine and Health Sciences, SU) in Cape Town (see map in Fig. 2, which depicts the Ukwanda Rural Clinical School, SU, and the sites where students are placed [23]).

### Characteristics of participants

The CIG comprised ten participants: two medical students, six clinician educators from Robertson District Hospital (generalist medical doctors and a postgraduate trainee in family medicine), the RCS academic director and the lead clinician educator (the district hospital family physician and main researcher). The family physician was the clinical head of the medical team of five generalist doctors and the LIC site’s lead clinician educator. The lead clinician educator and RCS academic director were the initiating researchers, and invited all three students and the six district hospital’s clinician educators to participate in the CIG. One of the three students allocated to the site opted to be transferred to the Worcester RCS site for the second half of the academic year and was not included in the CIG. One of the authors was not included as a CIG group member, as she did not share the work space of the LIC site and had no relationship to the LIC students and clinician educators.

The CIG concentrated on potential issues of power and hierarchy during the first meeting. The participants worked together for about 5 months at the onset of the CIG, which encouraged open discussion, shared ownership and trust during the inquiry process. The CIG agreed that the RCS academic director (the only participant not based at the district hospital, but had an oversight responsibility of the students and clinician educators at the LIC site) would facilitate the CIG meetings. The facilitator aimed at a genuine collaborative and democratic group process. All participants gave their informed consent.

### Co-operative inquiry group process (the intervention)

The CIG planned to meet during 2013, when the intervention should benefit the 2013 group of LIC students placed at Robertson Hospital. The focus and nature of this intervention would be decided upon by the CIG. Two cycles of the PAR process (action, observation, reflection, planning) were completed between June and October 2013 (see Table 1). During the two initial CIG meetings the CIG process and participant roles were explained. Student and clinician educators reflected on their learning experiences and expectations in the district hospital environment. The CIG reflected on this data their third meeting, where an educational intervention was planned. Finally, the CIG reflected on the intervention at their last meeting.

**Table 1:** Participatory action research process and data collection methods

PAR steps	Activities (timelines)	Participants	Data collection methods
Reflection and planning	CIG meetings 1 and 2 (19 June 2013 and 3 July 2013)	Two medical students One clinician educator RCS academic director Lead clinician educator	Transcription of audio-recordings made during the meetings
Action and observation	Data collection A (15 July 2013)	Three clinician educators Lead clinician educator	Focus group interview
Action and observation	Data collection B (26 July 2013)	Medical student who opted to leave the LIC site Independent interviewer	Individual interview
Reflection and planning	CIG meeting 3 (12 September 2013)	Two medical students Six clinician educators RCS academic director Lead clinician educator	Transcription of audio-recordings made during the meeting
Action and observation	Implementation of the agreed learning intervention during the final 6 weeks of the students’ placement at this LIC site (mid-September to October 2013)	Two medical students Six clinician educators Lead clinician educator	None
Reflection and planning	CIG meeting 4 (29 October 2013)	Two medical students Six clinician educators RCS academic director Lead clinician educator	Transcription of audio-recordings made during the meeting

*CIG* co-operative inquiry group, *PAR* participatory action research

### Documentation of the group process (data collection)

Data was collected using qualitative methods, including one focus group interview with clinician educators who could not attend the first two meetings, one individual interview and the transcribed audio recordings of the four CIG meetings.

### Building a consensus of the group’s learning (data analysis)

The transcripts were analysed using the six steps of data analysis as described by Creswell [24]. The qualitative data analysis software programme Atlas.ti (version 7.1.7) was used to identify categories and themes.

Data from the first two meetings and the interviews was analysed prior to meeting 3, during which the CIG validated the data. At meeting 4, the CIG reflected on the intervention and proposed interventions based on a consensus of what they had learned in this participatory process. Following the conclusion of the CIG process, all the data was analysed by the three authors. Two authors independently read the coded transcripts as generated by the first author with the assistance of Atlas.ti. A number of meetings were arranged in which the three authors discussed, clarified and reached consensus on the themes and categories that emerged from the data. Quotations were selected that best represented the perceptions of students, clinician educators, lead clinician educator and the academic director (the participants) in relation to the themes.

## Results

The CIG process was conducted over 5 months, during which the CIG participants became more aware of the challenges and opportunities inherent to the district hospital learning context. This allowed the CIG to design and reflect on a tailored teaching and learning intervention. This intervention centred on addressing the assessment-related student anxiety, as well as clarifying the supervising responsibilities within the team of clinician educators.

The CIG designed a structured learning programme for the final 6 weeks as the agreed intervention. These 6 weeks were used by the students prepare for their final exit examination. Each day was divided into two slots, with the morning period allocated towards self-study, and the afternoon dedicated to patient-centred clinical teaching activities with the lead clinician educator (the family physician). The afternoon’s teaching interaction and format was tailored to the students’ self-identified learning need for a particular discipline.

The results section is organised according to three main themes which emerged from the data analysis. These themes relate to three paradigm shifts experienced within this LIC site. Key reflections from before and after the intervention are presented for each theme. Translated quotations are presented in square brackets.

1. Shift from the traditional medical education approach to the transformative learning approach advocated for the 21^st^ century health professional
  This new learning approach was met with tension among students and clinician educators.
  - Initial reflection
    Students felt that adapting to the LIC model was challenging.
    *I had this idea that it will be very different and that there will be no specialists and that I will be much more responsible for my own learning; however, it was an even greater reality check on arrival … but one has discovered one’s own way of adjustment, one’s own learning style and what works. So clearly, I had no way of really anticipating what lay ahead.* (St3)
    Furthermore, the students experienced the final examination at the tertiary teaching hospital as a huge source of tension.
    *It’s (the examination) which creates a lot of tension amongst all of us … but I feel very unsure about how I will be able to cover all the work in the time available. To cover all those subjects in 2 weeks.* (St1)
    Clinician educators did not feel equipped to provide detailed academic input for students.
    *It is difficult for us … to give the nitty-gritty diagnoses … or different pathology we don’t see every day, but we can probably offer them a good practical idea on how to approach any patient.* (CE4, FG)
  - Reflection following intervention
  Students valued the intervention as time well spent, and felt better prepared, even if the assessment-related tension remained.
  *I thought it was an excellent idea. It worked really well for me. I think even with it I still felt anxious, I still have a lot of work, will I get through it. But we had a lot of study time, without it we would have been lost.* (St3)
  Clinician educators valued the role clarification: students should learn the theory and come to them for advice on the practical application of theory.
  *So it is better if you come to me with the theory and then we can see where we can meet (in practice).* (CE5)
2. Shift from teaching hospital context to district health system (DHS) context
  This new context challenged the traditional understanding of how learning may be structured.
  - Initial reflection
    The clinician educators and students agreed that the learning environment lacked a sense of structure.
    *I think there just needs to be a more formal learning structure for them in the hospital itself. … such as academic ward rounds …, which … is arranged once a week with a visiting consultant.* (CE3, FG)
    Furthermore, the clinician educators experienced uncertainty regarding the required learning outcomes.
    *We must be aware of what those learning outcomes are that they need; … sometimes I do also get the feeling that’s it’s sort of what’s there we teach them but maybe they need beyond that.* (CE3, FG)
    The findings also revealed that the clinician educators felt that they were teaching the students to become generalist doctors, and not specialists with detailed knowledge of uncommon problems.
    *I think in the end what it comes down to is we trying to teach them to be doctors, they walk out here with the ability to do the things that are expected of them as doctors. We [are not training] them to become……specialists…., we are training doctors to walk out sufficiently capable to treat a patient …* (CE1, FG)
  - Reflection following intervention
    Students valued the close correlation between the morning self-study topic and the afternoon discussions based on identified learning needs. The discussions were structured around patients and skills, in anticipation of the assessment ahead.
    *The afternoon sessions were structured, it was nice. We told (the lead clinician educator) in advance tomorrow we would like to discuss (name of subject). Then we came and we sat for two (to) two and a half hours and talked through it… So we were focused and I walked away and felt I did not waste my time. It was a very productive day, I studied the mornings and the afternoons were focused discussions.]* (St3)
    The students remain aware of the inherent security associated with the familiar, traditional learning structure. Students grew accustomed to the new learning environment and followed a different strategy to structure their own learning. This includes learning from patients, which is enhanced by the patient portfolio method.
    *It is the structure, I told you at the start of the year that it was a leap of faith for me to come here [to the longitudinal site] as I was used to the structure, of having tutorials on certain days, it is that sense of feeling safe in your comfort zone, because you know it is not like that here [at the longitudinal site] but I do not miss anything else besides [this feeling of structure.* (St3)
    The clinician educators believed that the intervention could inform the structuring of the teaching activities in the district hospital during the whole placement year.
    *I think it is a good model for the (whole) year (for) new students. To do it at the beginning of the semester and then you intensify it towards the end. Let’s say one day a week … it (provides) focus instead of scrambling through everything to help you learn, especially if you come with what you want to learn about, and then it is better.* (CE5)
    Taking ownership of own learning needs to be balanced with supporting guidance and monitoring of academic progress.
    *I think it is important to give (the students) guidance. (For example, during) the specialist outreach visits (visiting specialists), optimise the visits for the students, and use this opportunity for a positive learning opportunity.* (CE2)
    *I think all the (teaching and learning-related) suggestions will depend on someone monitoring (their implementation).* (St2)
    The clinician educators valued the role clarification facilitated by the PAR process, as they felt more at ease to know who was allocated to student supervision on a particular day.
    *What each and everyone’s role is, the family physician, the (clinician educators) … I think it is important for the students to know who they must involve where and to whom they can go.* (CE2)
    A renewed effort should be made for the team of doctors to familiarise themselves with the students’ study guide, with specific reference to the ‘list of common conditions’ per discipline (SU Rural Clinical School) and the requirements of the patient-related portfolios.
    *I think it is important to start at the basics and to show the student study guide (with learning outcomes) to each (clinician educator). This contains the outcomes that our students must attain each year … And we must know these outcomes.* (CE2)
3. Shift from block-based teaching to a longitudinal, integrated learning model
  Students displayed resourcefulness when navigating this new model of integrated experiential learning.
  - Initial reflection
    The students felt that repeated practical exposure enhanced their learning towards becoming comfortable in the doctor role.
    *[I believe that if you see something performed practical and then you do it and learn, then you recall it much better. Something which helps me is if you see it again and again … and to revisit the book afterwards and learn it and …. then you such a patient again and then you do it correct the next time.]* (St1)
    Furthermore, the students felt responsible for using available learning opportunities optimally.
    *I believe it depends on [which opportunities] you use to learn from. We have the opportunity to utilise more learning opportunities should we desire to do so.* (St3)
    Clinician educators regarded self-driven students as an important trait for success in the LIC model.
    *So now it does come down to [the] personality of the student, hopefully the ones that choose to do the longitudinal [model] have the personality that’s self-driven and they have the realisation that this is …their own onus to get the study work done.* (CE3, FG)
  - Reflection following intervention
    The students used their own initiative to involve other health professionals to help address their learning needs, when the clinical workload prevented the planned afternoon session from taking place (the designated clinician educator was not available).
    *There were some afternoons … the hospital (was) busy and you went and helped out where you can. It is part of the package. But I think there was a great deal of effort to support us. I know at the maternity ward sister went through some topics (such as the partogram) with us. That is important and that, so we involved other people as well.* (St2)
    A shift towards integrating work space-based learning and assessment in clinical disciplines is advocated.
    *Work-wise it is like that (integrated clinical experience), but it isn’t with the studying (for assessment) like that and that is the big problem.* (St2)
    *But I think longitudinal (the LIC model) must remain longitudinal. … I think from the university must look at other ways of evaluating the students because their anxiety is around the (discipline-specific evaluations). But I know in future it will be worked on.* (CE2)

## Discussion

This research describes the insights gained by a co-operative inquiry group on how to improve the learning experience of LIC students during their immersion in the rural district health services. The positive learning experiences shared by students and their clinician educators are juxtaposed to the constraints of the fast-paced environment, in which a structured learning schedule remains to be established. The CIG reflected on their experience of implementing a teaching and learning intervention and made suggestions on which practical strategies will enhance the available learning opportunities within this newly established LIC site. These strategies may also benefit other LIC sites in similar contexts.

Three global paradigm shifts were experienced in the micro-environment of this rural district hospital.

1. Shift from the traditional medical education approach to the transformative learning approach advocated for the 21^st^ century health professional
  The immersion of students in this relatively new learning context created tension among all the actors in this context. Much of this tension is centred on the misconception of what ‘academic knowledge’ is. In this study, participants understood academic knowledge as detailed textbook-based facts, which were perceived to be important for their final exit examination. Clinician educators felt challenged to meet the learning needs of the students, as they felt that their exposure to current theoretical training was dated. However, the findings of this study demonstrate how ‘academic knowledge’ can be gained in a different way through experiential learning when students manage their own patients under supervision.
2. Shift from teaching hospital context to district health system (DHS) context
  Implementing a LIC training model in the context of the DHS is challenged by the lack of a culture of clinical training experience. This culture of clinical training of the next generation of health workers is embedded in the traditional teaching hospital. However, the tertiary context does not support the development of generalism or exposure to rural health. The lack of teaching structure in the DHS may be a manifestation of the lack of strategic insight into what is needed in the DHS to create a more supportive learning environment [25].
  Most of the day-to-day clinical supervision of the LIC students is done by the medical officers and family medicine registrar, whose main job is clinical care, and not the family physician, who is seen as the lead clinician educator at the LIC site. The family physician is tasked with a number of roles, such as clinical governance and being a consultant to the primary care team, which limits the time spent on student supervision [26]. The absence of clear supervisory roles as well as a lack of perceived structure created tension and anxiety. The LIC students came from the traditional tertiary training environment with signposted learning opportunities (tutorials, academic ward rounds, lectures according to a pre-planned schedule). Better student preparation prior to immersion in this rural workplace-based setting may smooth this transition. Furthermore, the local clinician educators and supportive role players (other staff categories and management) require orientation to the expected learning outcomes, the interplay between their rural context and learning opportunities, and potential clinical teaching tools available to clinician educators to facilitate student learning [27, 28].
3. Shift from block-based teaching to a longitudinal, integrated learning model
  The longitudinal integrated clerkship requires a paradigm shift from the traditional Flexnerian training model [19]. A new system of learning techniques (such as reflection on learning and creating conversation platforms with clinician educators to address identified learning needs) is required in addition to more traditional learning opportunities [27, 28]. Students need to be self-driven in identifying opportunities; they also need to be able to adapt to the changing nature of the day-to-day clinical environment, which determines the availability of the clinician educators. Students are able to rely on interpersonal relationships when seeking guidance during learning opportunities in the work place, which is made possible by their longitudinal attachment to the clinical team (clinician educators and other health workers). These abilities to work well with others, being able to adapt to a new environment, to think critically, to solve problem and have the right attitude are all skills required by students when entering the work place (during the LIC placement as well as during future employment, once qualified) [29].

### Strengths and weaknesses of the participatory action research method

This paper demonstrates the value of using the PAR method to identify how to develop a learning intervention tailored to the individual students’ and clinician educators’ identified needs in a LIC site. Participatory action research was described previously for health professions education at undergraduate, postgraduate and continuous professional education levels [30–32]. The LIC model, however, lends itself particularly well to the PAR method, as the longitudinal relationship facilitates the development of trust between the students and clinician educators. Furthermore, the experiential learning nature of the LIC model links closely with the PAR process of reflection in and on action.

The findings of this paper underscore the voices of the medical officers in their role as clinician educators at this rural district hospital. This represents a unique angle in terms of the existing research performed during the first 5 years of SU’s Rural Clinical School, as previous research focused only on the perceptions of the students and the regional hospital’s clinician educators [27, 33].

The PAR method contributed to creating an action learning set (the co-operative inquiry group) in which all role players engaged actively in the creation of the intervention which addressed the identified learning need (the intervention was timely, specific and tailored to the actors and the setting). Potential conflict among group members (group dynamics and power gradients) was avoided in this study as far as possible; attention to group ownership of the inquiry process was emphasised, as well as the ability of the group to reflect on their individual and collective experience, and allowing for sufficient trust and democracy to take practical action based on the group’s reflection.

### Key recommendations

Based on the group’s final consensus, the success of a teaching and learning strategy to address local learning needs largely depends on a collaborative approach to align learning experiences to the expected outcomes. It is important to create a sense of a structured programme based on a shared understanding of the expectations and learning outcomes. An ongoing conversation between the university, clinician educators and students regarding these expected outcomes should be nurtured. Attention should be given to prepare students for the LIC context, in which different signposts for learning opportunities exist.

Table 2 demonstrates how structured and tailored learning activities may be balanced in a LIC site. A solid framework for each week’s learning activities will help create clarity around roles and opportunities, whereas tailored interventions could be used towards addressing individual learning needs.

**Table 2:** Balancing structured and tailored learning activities in a LIC site

Degree of structure	Activities	Learning
Structured	Weekly roster of activities (e.g., clinical activities, specialist outreach visits and scheduled assessments)	Planned, sheltered teaching and learning time (e.g., time with family physician or visiting specialist)
Individualised/ Tailored	Determined by variability of patients presenting to emergency centre or primary care clinic, especially if there is a self-identified need of student to see a patient with a specific condition required for patient portfolio (according to the prescribed list of ‘common conditions’); Also determined by the variability of context, e.g., supervisory capacity of clinician educators during workload fluctuations.	Ad-hoc learning activity to address individual’s learning needs (e.g., individual student’s learning gap identified in shared patient consultation or book-learning for assessment)

This study furthermore highlights the value of the PAR paradigm, which enables the local LIC participants to co-develop teaching and learning interventions for this training model. The authors recommend that action learning sets should be developed at all the LIC sites where learning outcomes are negotiated. It is also recommended that new members (students and clinician educators) of the LIC action learning sets are orientated to the CIG method and learning outcomes.

## Conclusion

By reflecting on their experiences of learning via the CIG method, students and clinician educators were able to shape a joint approach towards improving learning at this newly established LIC site in a rural South African setting.

### Ethics approval

Approval for this study was obtained from SU’s Health Research Ethics Committee (N13/04/048) and from the Provincial Health Research Committee, Western Cape Government Health, South Africa (RP 91/2013).

## Abbreviations

CE: clinician educator
CIG: co-operative inquiry group
DHS: district health system
FG: focus group
LIC: Longitudinal Integrated Clerkship
PAR: participatory action research
RCS: the Ukwanda Rural Clinical School
St: Student
SU: Stellenbosch University
